# Wee1 kinase inhibitor adavosertib with radiation in newly diagnosed diffuse intrinsic pontine glioma: A Children’s Oncology Group phase I consortium study

**DOI:** 10.1093/noajnl/vdac073

**Published:** 2022-05-20

**Authors:** Sabine Mueller, Tabitha Cooney, Xiaodong Yang, Sharmistha Pal, Ralph Ermoian, Amar Gajjar, Xiaowei Liu, Komal Prem, Charles G Minard, Joel M Reid, Marvin Nelson, Daphne Haas-Kogan, Elizabeth Fox, Brenda J Weigel

**Affiliations:** Department of Neurology, University of California, San Francisco, San Francisco, California; Department of Pediatrics, University of California, San Francisco, San Francisco, California; Department of Neurosurgery, University of California, San Francisco, San Francisco, California; Dana-Farber/Boston Children’s Cancer and Blood Disorders Center, Harvard Medical School, Boston, Massachusetts; Department of Neurosurgery, University of California, San Francisco, San Francisco, California; Department of Radiation Oncology, Brigham and Women’s Hospital, Dana-Farber Cancer Institute, Boston Children’s Hospital, Harvard Medical School, Boston, Massachusetts; Department of Radiation Oncology, University of Washington Medical Center, Seattle, Washington; St. Jude Children’s Research Hospital, Memphis, Tenesse; Children’s Oncology Group, Monrovia, California; Molecular Pharmacology and Experimental Therapeutics, Mayo Clinic College of Medicine, Rochester, Minnesota; Institute for Clinical and Translational Research, Baylor College of Medicine, Houston, Texas; Molecular Pharmacology and Experimental Therapeutics, Mayo Clinic College of Medicine, Rochester, Minnesota; Children’s Hospital Los Angeles, Radiology, Keck USC School of Medicine, Los Angeles, California; Department of Radiation Oncology, Brigham and Women’s Hospital, Dana-Farber Cancer Institute, Boston Children’s Hospital, Harvard Medical School, Boston, Massachusetts; St. Jude Children’s Research Hospital, Memphis, Tenesse; Department of Pediatrics, University of Minnesota, Minneapolis, Minnesota

**Keywords:** adavosertib, DIPG, radiation-sensitization, toxicity, Wee1

## Abstract

**Background:**

Children with diffuse intrinsic pontine gliomas (DIPG) have a dismal prognosis. Adavosertib (AZD1775) is an orally available, blood-brain barrier penetrant, Wee1 kinase inhibitor. Preclinical efficacy against DIPG is heightened by radiation induced replication stress.

**Methods:**

Using a rolling six design, 7 adavosertib dose levels (DLs) (50 mg/m^2^ alternating weeks, 50 mg/m^2^ alternating with weeks of every other day, 50 mg/m^2^, then 95, 130, 160, 200 mg/m^2^) were assessed. Adavosertib was only given on days of cranial radiation therapy (CRT).The duration of CRT (54 Gy over 30 fractions; 6 weeks) constituted the dose limiting toxicity (DLT) period. Endpoints included tolerability, pharmacokinetics, overall survival (OS) and peripheral blood γH2AX levels as a marker of DNA damage.

**Results:**

A total of 46 eligible patients with newly diagnosed DIPG [median (range) age 6 (3–21) years; 52% female] were enrolled. The recommend phase 2 dose (RP2D) of adavosertib was 200 mg/m^2^/d during days of CRT. Dose limiting toxicity included ALT elevation (n = 1, DL4) and neutropenia (n = 1, DL7). The mean T_max_, T_1/2_ and Cl_p_ on Day 1 were 2 h, 4.4 h, and 45.2 L/hr/m^2^, respectively. Modest accumulation of adavosertib was observed comparing day 5 versus day 1 AUC_0-8h_ (accumulation ratio = 1.6). OS was 11.1 months (95% CI: 9.4, 12.5) and did not differ from historical control.

**Conclusion:**

Adavosertib in combination with CRT is well tolerated in children with newly diagnosed DIPG, however, compared to historical controls, did not improve OS. These results can inform future trial design in children with high-risk cancer.

**Key Points**

1. Adavosertib (200 mg/m^2^) administred on days of focal RT (54 Gy) was established as the RP2D.
2. Adavosertib in combination with focal RT is well tolerated in newly diagnosed children with DIPG.

**Importance of the Study**

Targeting DNA damage or replication stress is an important therapeutic avenue for enhanging radiation sensitivity of cancer. This study, conducted in patients with newly diagnosed diffuse intrinsic pontine glioma (DIPG), was the first trial to combine a specific Wee1 inhibitior, adavosertib, with radiation therapy. The study defined the recommended phase 2 dose of adavosertib in combination with cranial irradiation (54 Gy, 30 fractions), highlighting the importance of building on standard of care therapy to conduct early phase studies in children with the highest risk cancers. This approach was feasible and generated important pharmacological information to build on for future studies.

Diffuse intrinsic pontine glioma (DIPG) portends a dismal prognosis in children, with virtually no long-term survivors.^1^ Despite a significant increase in our understanding of the underlying biology of these tumors, radiation treatment remains the only known therapy for prolonging life. Thus, agents that effectively synergize with radiation may greatly impact survival.

Wee1 kinase is a regulator of G_2_-M cell cycle progression.^2^ In response to DNA damage, several mediators including ataxia-telangiectasia (ATM) and rad3-related protein (ATR) activate Chk1, which in turn activates Wee1. Activated Wee1 phosphorylates CDC2, inhibiting its function. In normal cells and most cancer cells, increasing levels of inactivated, phosphorylated CDC2 lead to G_2_ checkpoint activation, preventing damaged cells from entering mitosis prior to DNA repair. Without a proficient G_2_ checkpoint, cells progress through the cell cycle with damaged DNA, and ultimately succumb to fatal mitosis.^3–6^ Thus, it was suspected that targeting G_2_ by Wee1 kinase inhibition may increase the tumor specific toxicity of radiation induced DNA-damage. Indeed, this has been shown in some cancers including breast cancer.^7,8^ Furthermore, prior studies implicated Wee1 kinase with outcome in adult glioblastoma, and Wee1 expression levels correlated inversely with overall survival (OS).^3^ Previously, we have shown that Wee1 is overexpressed in pediatric high-grade compared to pediatric low-grade gliomas with the highest level noted in a patient derived DIPG cell line.^9^

Adavosertib is a specific, ATP competitive and highly selective inhibitor of the Wee1 kinase.^10,11^ Through Wee1 inhibition, adavosertib induces G_2_ checkpoint escape and, thus, enhances the apoptotic effects of DNA-damaging agents or radiotherapy.^12,13^ Using orthotopic *in vivo* models of adult glioblastoma multiforme (GBM), Mir *et al.* demonstrated that combining radiation therapy with adavosertib significantly improved survival.^3^ Using *in vitro* clonogenic survival assays, we found adavosertib exhibited dose-dependent anti-proliferative effects in multiple pediatric glioma cell lines including a DIPG derived cell line, and this effect was enhanced by radiation.^9^ We and others have found that adavosertib in combination with radiation therapy prolongs survival in murine models of pediatric high-grade glioma including DIPG.^9,14^

Adavosertib has been evaluated as monotherapy as well as in combination with standard chemotherapy regimens and has shown promise in combination with cisplatin in cisplatin resistant ovarian cancer.^15^ The first pediatric study assessed adavosertib in combination with irinotecan in relapsed solid tumors.^16^ However, adavosertib has not been assessed in combination with CRT in children.

Given the underlying mechanism of action, blood brain barrier penetration of adavosertib^17,18^ and our preclinical efficacy in models of DIPG,^9^ we aimed to evaluate the safety and antitumor activity of adavosertib when given concurrently with CRT in children with newly diagnosed DIPG in a phase I clinical trial (ADVL1217) conducted by the Children’s Oncology Group (COG) Phase I consortium.

## Materials and Methods

### Study Design and Participants

The study had three primary aims: (1) to estimate the maximum tolerated dose (MTD) or establish the recommended phase 2 dose (RP2D) and schedule of the Wee1 inhibitor adavosertib (AZD1775; MK-1775) administered concurrently with CRT in children with newly diagnosed DIPG, (2) to define and describe the toxicities of adavosertib, and (3) to characterize the pharmacokinetics (PK) of adavosertib when given concurrently with CRT in this patient population. Secondary endpoints included defining the antitumor activity of adavosertib, and assessing the biologic activity by measurement of γH2AX in peripheral blood mononuclear cells (PBMCs).

Patients older than 3 years and younger than or equal to 21 years with newly diagnosed DIPG were eligible. DIPG was defined as tumors with a pontine epicenter and diffuse involvement of the pons. Patients with brainstem tumors that did not meet these criteria were eligible if the tumor was biopsied and proven to be anaplastic astrocytoma, glioblastoma, gliosarcoma, diffuse midline glioma with H3K27M mutation, or anaplastic mixed glioma. Patients could not have received any anti-cancer therapy, except surgery, prior to enrollment. Patients with disseminated disease were excluded. Patients must have been able to swallow capsules.

Patients were required to have a Karnofsky (those older than 16 years) or Lansky (those younger than 16 years) performance score of at least 50. Patients had to have adequate organ function including bone marrow (absolute neutrophil count ≥1000 cells per μL and transfusion independent platelet count ≥100 000 cells per μL), renal (normal serum creatinine for age), hepatic (total bilirubin ≤1·5 times upper limit of normal (x ULN), alanine aminotransferase ≤3·0 × ULN, aspartate aminotransferase ≤3·0 × ULN), neurologic (seizure disorder eligible if well controlled on nonenzyme inducing anticonvulsant; nervous system disorder ≤ grade 2 CTCAE v5 with exception of tendon reflex), and cardiac (QTc ≤ 480 ms) function. Patients receiving corticosteroids were eligible. Patients who were pregnant, breastfeeding or had an uncontrolled infection were not eligible.

The protocol was reviewed and approved by the Cancer Therapeutics Evaluation Program (CTEP) of the National Cancer Institute (NCI) and the institutional review boards of all participating sites. The study was conducted in accordance with the principles of the World Medical Association Declaration of Helsinki. Informed consent and child assent, when appropriate, were obtained from all participants and/or parents or legal guardians.

Source documents were verified on site at a regular basis for all patients enrolled at COG phase I consortium sites. This study is registered with ClinicalTrials.gov (NCT01922076).

### Procedures

Patients received focal CRT at the current standard dose of 54 Gy in 180 cGy fractions, Monday through Friday for a total of 30 fractions over 6 weeks, with a rest period on Saturday and Sunday of each week. Adavosertib was given orally, once daily following the scheduled CRT fraction. If CRT was delayed because of holiday or other logistical reasons, patients were advised to hold adavosertib.

The starting dose of adavosertib was 50 mg/m^2^ given on days of CRT during weeks 1, 3 and 6 of CRT. Treatment was first escalated by increasing the number of days adavosertib was given during weeks one through six of CRT (50 mg/m^2^/day Monday through Friday alternating weeks; 50 mg/m^2^/day Monday through Friday alternating with weeks of every other day (Monday, Wednesday, Friday); 50 mg/m^2^/day Monday through Friday). Treatment was then escalated by increasing the dose of adavosertib (95, 130, 160, 200 mg/m^2^ per dose).

Adverse events (AE) were graded according to the Common Terminology Criteria for Adverse Events version 5.0. Hematological DLTs were defined as grade 3 thrombocytopenia or grade 4 neutropenia; nonhematologic DLT as any grade 3 or 4 AE possibly, probably, or definitely attributable to adavosertib with the exception of: grade 3 nausea or vomiting that resolved within 3 days, grade 3 liver enzyme elevation that returned to baseline or levels that met initial eligibility criteria within 7 days, grade 3 or 4 fever of less than 5 day duration, grade 3 infection of less than 5 days duration, or grade 3 serum mineral or electrolyte disturbances that resolved with oral supplementation. We also defined nonhematologic DLT as any grade 2 nonhematological toxicity attributable to adavosertib that persisted for ≥7 days and was considered sufficiently medically significant or sufficiently intolerable by patients that it required interruption of adavosertib for 7 days. Further, any AEs that interrupted planned radiation for 5 consecutive fractions or 10 fractions total or resulted in a treatment delay of adavosertib of more than 7 days were also considered a DLT. CRT was not interrupted for adavosertib related DLTs unless clinically indicated.

### Pharmacokinetic Analyses

Blood samples (2–3 mL) were collected in dipotassium EDTA tubes during Cycle 1 at baseline (all patients) Day 1 (patients ≥20 kg: 1, 2, 4, 6, 8 and 24 h after dose), Day 4 (patients ≥20 kg: predose), and Day 5 (all patients: predose, 1, 2, 4, 6, and 8 h after dose). Adavosertib plasma concentrations were measured by hydrophilic interaction liquid chromatography coupled with tandem mass spectrometry, as previously described.^19^

Pharmacokinetic parameters were calculated using noncompartmental methods^20^ using Phoenix^®^ WinNonlin^®^ Version 8.1 (WinNonlin version 8.1.03530, Pheonix 64; Pharsight Corporation, Mountain View, CA). The AUC over the 24 h dosing interval (τ) on Day 5 was calculated by trapezoidal approximation. Since a 24 h blood sample was not drawn, the 24 h plasma concentration after adavosertib administration was estimated to be equivalent to the predose concentration based on the assumption that steady-state was reached on day 5. Adavosertib accumulation was calculated as the ratio of day 5 AUC_0-8h_: day 1 AUC_0-8h_. Oral steady-state clearance (Cl_SS_/F) was calculated using the equation, Cl_SS_ = Dose/AUC_τ_.

### Efficacy Analyses

Disease assessments were performed at baseline prior to start of CRT, within 3–4 weeks after completion of CRT, and then every 3 months. Response assessment was determined by changes in size using the maximal 2-dimensional cross-sectional tumor measurements, T × W (product of the longest diameter of the tumor [width (W)] and its longest perpendicular diameter [transverse (T)], using either T1 or T2 weighted images, similar to historical controls.^21,22^ We defined stable disease (SD) as failing to meet criteria for either partial response (PR), complete response (CR) or progressive disease (PD). PD was defined as any appearance of new lesion(s) or an increase of 25% or more in the sum of the product of the perpendicular diameters of the target lesions. PR was defined as a decrease of 50% or more in the sum of the products of the two perpendicular diameters of the target lesion. Two objective determinations of disease status, by MRI, obtained on two consecutive determinations, separated by at least a 4-week time period, were required to determine the patient’s overall best response. Imaging was reviewed centrally for 41 response-evaluable patients.

### Pharmacodynamic Analyses

The expression of γH2AX in PBMCs was measured pre and posttreatment using flow cytometry. Effort was made to obtain two baseline (pretreatment) samples on two different days, with the first occurring within 7 days prior to CRT. Serial samples were then collected on days 1 and 5 of treatment, 6–8 hours after each dose, and day 8 prior to the day’s dose. Blood samples of 1 mL were drawn into heparinized containing tubes and transferred to a Smart Tube (Thermo Fisher) followed by a 30 min incubation period in a 37°C water bath for activation per manufacture’s guidelines. Samples were stored at −80°C until shipment.

The frozen Smart Tubes were thawed in a 10–12°C water bath for 20 min before the red blood cells were lysed and intact cells were fixed in 90% methanol and stored at −20°C overnight. Briefly, the methanol fixed cells were washed and blocked in staining buffer (1X PBS containing 0.5 mg/ml BSA) containing TruStain FcX^TM^ (BioLegend) for 10 min at room temperature. Next, cells were incubated with FITC-conjugated anti-γH2AX Ser-139 antibody (BioLegend) for 1 h, washed and DNA stained with propidium iodide (2 μg/ml) before data colection by flow cytometry on CytoflexS. Percent positive γH2AX blood cells was determined by FlowJo software and described as fold-change over baseline for each patient.^16^

### Statistical Analysis and Design

A rolling six design was used to estimate the MTD or RP2D. Overall survival (OS) was estimated using the Kaplan Meier method including 95% confidence intervals (CI). OS was calculated as the time (in months) from enrollment until date of death. Patients who did not die were censored at last evaluation date. Progressive disease was based on central imaging review. If the patient did not have PD or death, then the patient was censored for the event at the last evaluation date. OS is compared with an historical cohort of patients which cumulatively includes DIPG patients from ACNS0126^23^ and ACNS0222^22^ using a log-rank test. PK parameters were analyzed with summary statistics, including means, and standard deviations.

## Results

### Patient Population

Forty-six patients, median age of 6 years (range 3 to 21 years), were enrolled from September 2013 to August 2018 across 17 sites. All 46 were eligible; 39 were fully evaluable for toxicity. Seven patients were inevaluable for toxicity because they did not receive at least 85% of the planned dose. Table 1 outlines patient characteristics for all eligible patients. A biopsy for diagnostic confirmation was not mandated but 14 participants underwent biopsy prior to enrollment. Of these, nine were diagnosed as H3K27 mutant glioma. At the time of this study routine examination of the H3K27M status was not yet fully implemented and therefore the status of H3 was unknown in some patients based on detailed pathology review (Table 1).

**Table 1.** Patient characteristics for eligible patients (n = 46)

Characteristic	Number (%)
Age (years) Median Range	6 3–21
Sex Male Female	22 (48) 24 (52)
Race White Asian America Indian or Alaska native Black or African American Unknown	28 (61) 4 (9) 1 (2) 6 (13) 7 (15)
Ethnicity	
Non-Hispanic Hispanic Unknown	37 (81) 7 (15) 2 (4)
Diagnosis	
H3 mutant diffuse midline glioma	9
Anaplastic astrocytoma, grade 3*	4
Glioblastoma* No biopsy	1 32
Prior therapy with steroids	13

*Molecular data not available.

### Toxicity

Adavosertib was escalated in cohorts of patients by dose frequency from 50 mg/m^2^/day Monday through Friday alternating weeks to 50 mg/m^2^/day Monday through Friday on days of CRT every week, then in cohorts of patients by dose, 95 mg/m^2^ to 200 mg/m^2^ per dose, using the rolling six design.^24^

One of six patients treated at dose level 4 (95 mg/m^2^/day Mon-Fri, during weeks 1–6) experienced dose limiting elevation in ALT (grade 3) that persisted for more than 7 days. In the six patients fully evaluable for toxicity at the highest dose level 7 (200 mg/m^2^/day Mon–Fri, during weeks 1–6) one experienced a DLT (grade 4 neutropenia). The study completed accrual to the dose finding cohorts. Table 2 shows grade ≥ 3 AEs associated with adavosertib for each dose level.

**Table 2.** Patient-courses with higher grade toxicities

Toxicity type	Number of patient-course with grade >= 3, # (%)							
	All dose levels (N = 89)	Dose level 1 (N = 14)	Dose level 2 (N = 11)	Dose level 3 (N = 15)	Dose level 4 (N = 15)	Dose level 5 (N = 13)	Dose level 6 (N = 11)	Dose level 7 (N = 10)
Lymphocyte count decreased	16 (18)	1 (7)	4 (36)		2 (13)	2 (17)	2 (18)	5 (50)
White blood cell decreased	5 (6)						1 (9)	4 (40)
Neutrophil count decreased	4 (4)						1 (9)	3 (30)
Anemia	3 (3)							3 (30)
Weight gain	2 (2)			1 (7)				1 (10)
Alanine aminotransferase increased	1 (1)				1 (6)			
Dizziness	1 (1)	1 (7)						
Febrile neutropenia	1 (1)							1 (10)
Headache	1 (1)	1 (7)						
Hypocalcemia	1 (1)			1 (7)				
Lung infection	1 (1)				1 (6)			

Includes AEs possible, probable or definitely related to adavosertib

### Pharmacokinetics of Adavosertib in Children with Newly Diagnosed DIPG

**Figure 1.**
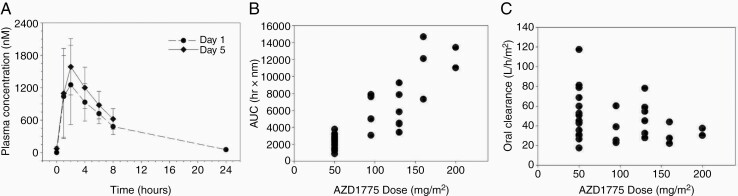
(A) Mean (SD) adavosertib plasma concentrations versus time profile on Day 1 (closed circles, n = 3) and Day 5 (closed diamonds, n = 6) for patients treated at the RP2D (200 mg/m^2^/day, Mon–Fri). (B) Graph of Day 1 AUC_0-∞_ versus dose as a method to estimate systemic exposure. (C) Graph of oral clearance (CL/F) adjusted for body surface area versus dose. SE: standard error.

Adavosertib pharmacokinetics were characterized in 40 patients (N = 34 patients ≥20 kg on Day 1 and N = 40 patients on Day 5) on the first week of daily ×5 treatment. The median age of this subset was 6 years (IQR = 3, range 3–21 years). The results are summarized in Table 3. Plasma concentration-time curves for patients treated at the RP2D are shown in Figure 1A. The PK parameters (mean ± standard deviation) on day 1 demonstrated T_max_ was 2 ± 1 h, half-life was 4.4 ± 1.1 h. Maximum plasma concentration (Cmax) and area under the concentration x time curve (AUC) appeared to increase in proportion to dose, as illustrated for AUC_0-∞_ in Figure 1B. The oral clearance was 45.2 ± 21.3 L/hr/m^2^ and appears to be lower at the highest dose levels (Figure 1C), however, the limited data available at those dose levels makes is difficult to assess the factors that might have contributed to those low values. CL/F was lower in females compared to males (39.2 L/h/m^2^ versus 50.9 L/h/m^2^), however this did not reach statistical significance (*P* = .12, independent two-sample t-test). Children younger than age 12 had higher mean CL/F compared to children age 12 or older however this was not statistically significant and limited by the small sample number in the older age cohort (46.0 L/h/m^2^ versus 39.5 L/h/m^2^; *P* = .29 Satterthwaite t-test for unequal variances). Following 5 days of daily administration, modest accumulation of adavosertib was observed based on comparison of the day 1 versus day 5 AUC_0-8h_. The median (range) accumulation ratio was 1.2 (range 0.6–2.2) (Table 3).

**Table 3.** PK summary of adavosertib

	Day 1								Day 5							
Dose Level	# pts	T_max_ (hrs)	C_max_(nM)	C_24h_ (nM)	Half-life (hr)	AUC_0-8h_ (nM•hr)	AUC_0- ∞_(nM•hr)	CL/F (L/ hr/m^2^)	# pts	T_max_ (hrs)	C_max_(nM)	C_24h_ (nM)	AUC_0-8h_ (nM•hr)	AUC(nM•hr)	CL_ss_ (L/hr/ m^2^)	R, AUC_0-8h_, D5/ D1
**(mg/ m** ^**2**^ **/d)**																
50	18	1.9 ± 0.9	356 ± 109	17.6 ± 29.8 (n = 14)	4.2 ± 1.3	1420 ± 438	2220 ± 800	51.1 ± 24.6	18	1.9 ± 0.9	422 ± 170	12.3 ± 7.8 (n* = 17)	1649 ± 654	2452 ± 1037	47.9 ± 23.2	1.21 ± 0.42
95	6	1.8 ± 0.4	869 ± 235	26.1 ± 18.6 (n = 5)	4.5 ± 1.2	3362 ± 850	6034 ± 1978	35.0 ± 14.5	6	1.9 ± 0.5	933 ± 351	19.2 ± 8.5 (n* = 5)	4012 ± 1809	5735 ± 2720	39.2 ± 17.2	1.16 ± 0.27
130	6	2.3 ± 0.8	821 ± 410	19.3 ± 6.9	4.1 ± 0.5	3731 ± 1583	5869 ± 2256	49.5 ± 18.5	6	2.3 ± 0.8	994 ± 467	49.8 ± 22.7	4484 ± 1770	7215 ± 2760	41.6 ± 19.8	1.22 ± 0.13
160	3	2.6 1.1	1274 ± 705	54.9 ± 28.0	4.8 ± 1.0	6689 ± 2738	11365 ± 3732	31.2 ± 11.3	5	2.2 ± 1.1	1690 ± 670	64.5 ± 37.8 (n* = 4)	8468 ± 2362	12802 ± 1804 (n* = 4)	25.6 ± 3.3 (n* = 4)	1.44 ± 0.19
200	2	2.5 ± 2.1	1654 ± 1051	78 ± 42.5	5.6 ± 1.3	6904 ± 2758	12217 ± 1700	33.9 ± 5.2	5	2.6 ± 1.4	2020 ± 780	100.6 ± 88.4	9633 ± 4222 (n* = 4)	16398 ± 7757	33.4 ± 25.8	1.64 ± 0.61

Note: CL/F, oral clearance; CL_ss_, steady-state clearance; R, accumulation ratio.

*Indicates number of patients for which data was available if different then listed under # pts.

Pharmacodynamic effects of adavosertib when given in combination with focal radiation therapy in children with newly diagnosed DIPG.

Specimens from 42 patients were submitted and sufficient for pharmacodynamic analysis. Baseline samples (n = 26) showed significant intrapatient differences and compared to baseline there were no significant changes γH2AX in PBMCs at any dose level (see supplemental Table 1).

### Clinical Activity of Adavosertib

**Figure 2.**
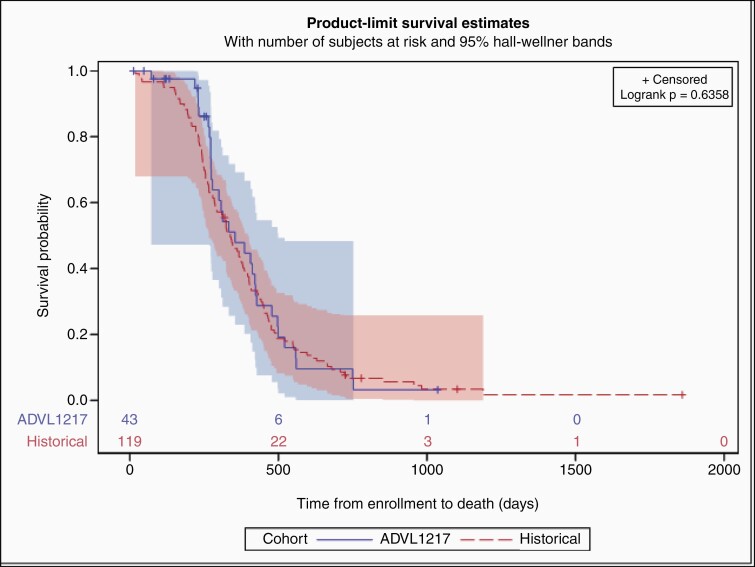
Depicted is the Kaplan Meier analysis with 95% Hall Wellner Band of patients enrolled in ADVL1217 compared to historical control data from COG (Log-rank *P* = .64).

Forty-one patients were evaluable for objective response with a median follow-up time of 354 days (time to death or last evaluation). Among those, 33 (80.5%) had SD as best overall response, the remaining 8 (19.5%) had PD. Analysis of dose-response relationship was limited by small sample size; there was no significant difference in best response by dose level (Fisher exact *P* = .82). Further, there was no significant difference in median OS in this study (11.8 months (95% Confidence interval (CI): 9, 13.9)) compared to historical control of 11.1 months (95% CI: 9.4, 12.5; *P* = .83) (Figure 2).

## Discussion

This is the first clinical trial of adavosertib in combination with CRT in pediatric patients with newly diagnosed DIPG. The study was based on preclinical data from our group and others showing efficacy of the combination of adavosertib and radiation therapy in pediatric high grade glioma models.^9,14^

Our results show that this combination was generally well tolerated in children with newly diagnosed DIPG. No MTD was reached. The RP2D defined as the highest dose level evaluated was adavosertib 200 mg/m^2^/day, daily Monday-Friday with standard fractionated CRT of 54 Gy. We did not escalate adavosertib higher than the 200 mg/m^2^/day dose level because a trial in adults with newly diagnosed glioblastoma, a similar schedule of adavosertib 200 mg/day (approximately 115 mg/m^2^/day) in combination with temozolomide was associated with significant toxicity.^25^ In our study, adavosertib in combination with standard dose radiation therapy was well tolerated. DLTs occurred at dose level 4 in one patient who experienced a grade 3 ALT elevation that did not recover to baseline within 7 days and one patient experienced grade 4 neutropenia at dose level 7. The only other known study of adavosertib in children, a phase I trial of adavosertib in combination with irinotecan for relapsed or refractory solid tumors (NCT02095132) defined the MTD as adavosertib 85 mg/m^2^/dose in combination with irinotecan 90 mg/m^2^/dose administered orally daily for 5 days on a 21 day cycle.^16^

The pharmacokinetics of adavosertib observed in this trial were consistent with those observed for pediatric patients in the previous COG trial.^16^ Peak plasma concentrations were observed 2 h after drug administration, the median half-life was 4.4 h and modest accumulation was observed over the 5 day course of treatment. The median BSA-adjusted plasma clearance value of 45.2 L/hr/m^2^ observed in this trial was comparable to the value of 32.5 L/hr/m^2^ in the earlier study. Adavosertib pharmacokinetics have been characterized in several adult Phase I trials.^26–29^ In general, higher adavosertib peak concentrations were acheived sooner (T_max_, 2 h versus 4 h) and the elimination half-life was shorter (t_1/2_, 4.4 h versus 9–11 h) in pediatric patients as compared to adult patients. However, AUC_0-8 h_ was similar in children and adults at equivalent doses. A recent report of a food-effect study noted that the mean oral clearance of adavosetib in fasting adults was 40.85 L/hr which was similar to the mean oral clearance of 46.9 L/hr in children found in this study.

Prior pharmacodynamic studies of adavosertib included pre and posttreatment tumor core or skin punch biopsies for inhibition of CDK1 phosphorylation or induction of γH2AX signifying enhanced DNA damage.^26,27,30^ In our preclinical studies, treatment with adavosertib led to increased expression of γH2AX in tumor tissue from treated mice compared to control animals. More recently, Cole et al. examined peripheral blood mononuclear cell γH2AX induction by flow cytometry to the combination of adavosertib with irinotecan, but could not determine the effects of adavosertib alone.^16^ Similarly, we examined peripheral blood mononuclear cell (PBMCs) γH2AX induction by flow cytometry, but could find no significant changes compared to baseline or correlations with dose levels.

A secondary objective of this study was to assess objective antitumor activity. An important consideration of any systemically administered agent for DIPG is effective tumor penetration necessary to improve outcomes. A phase 0 trial of adavosertib in first-recurrence adult glioblastoma demonstrated good brain tumor penetration following a single 400 mg dose.^18^ However, CNS penetration for brainstem gliomas might differ since these are often nonenhancing tumors. There are now ongoing phase 0 or target validation studies in DIPG that assess drug concentration and downstream effects directly in tumor tissue. These designs will more appropriately allow for direct assessments of blood-brain-tumor penetration of novel agents into brainstem tumors but this has not been completed for adavosertib in this patient population.

Key limitations of this study include, limited availability of detailed molecular profiling of DIPG tumor tissue of enrolled participants, as well as the trial design to administer adavosertib during radiation without continuation in a maintenance phase after radiation. In addition, since this was the first trial of adavosertib in combination with radiation, the starting dose was conservative and seven dose levels were evaluated without exceeding the MTD.

Since the initiation of this trial in 2012, the community has learned a tremendous amount about the underlying biology of DIPGs. We now understand that the H3K27M mutation occurs in combination with various partner mutations that might influence outcome as well response to any new therapy regimens.^31–33^ Based on the availability of 14 pathology results, the majority of tumors tested positive for the H3K27M, which is aligned with published reports, however detailed information on partner mutations was not available. Obtaining biopsies in children with DIPG with subsequent detailed profiling using next generation sequencing technologies, such as whole exome sequencing or RNA seq, is becoming standard in most centers with dedicated pediatric neuro-oncology programs. Studies have shown that this can be achieved in a safe manner in a multi-institutional setting.^34,35^ More in depth understanding of the molecular profile might have supported further investigations of this combination in a specific subset of DIPGs.

In this trial the OS was not superior compared to other historical trials conducted through COG or compared to other published reports.^1,36^ It is unlikely that radiosensitization with a novel agent such as adavosertib, without ongoing maintenance therapy, will control these tumors long-term. Therefore, it is critical to develop rational combinations with CNS penetrant chemotherapy agents that show synergy with adavosertib.

The development of novel therapeutic strategies for a disease like DIPG will also need to include assessments that allow for the determination as to why therapies fail despite encouraging preclinical data. In this study, we aimed to asses γH2AX as a marker of DNA damage in the PBMCs but no correlation was found. In future studies, it will be critical to understand the effects on tumor cells of these therapies and, therefore, new strategies such as cerebrospinal fluid assessments of circulating tumor cells or on treatment biopsies might be needed to improve outcomes for these tumors.

In conclusion, in children and adolescents with newly diagnosed DIPG the RP2D of adavosertib is 200 mg/m^2^/day in combination with standard fractionated focal CRT of 54 Gy. This can serve as a baseline for future studies aiming to use adavosertib or other radiation sensitizing agents in combination with CRT.

## Supplementary Material

vdac073_suppl_Supplementary_Table_S1
