# Supplementary material for: Wee1 kinase inhibitor adavosertib with radiation in newly diagnosed diffuse intrinsic pontine glioma: A Children’s Oncology Group phase I consortium study
Source: Neurooncol Adv. 2022 May 20;4(1):vdac073. doi: 10.1093/noajnl/vdac073 (PMC9209747; doi:10.1093/noajnl/vdac073)
Supplement: vdac073_suppl_Supplementary_Table_S1 [file vdac073_suppl_supplementary_table_s1.docx]

**Supplemental Table1**: γ-H2AX positive cells in peripheral blood cells upon adavosertib therapy as determined by flow cytometry. N/A represents data not available.

| Dose Level | Patient # | Pre-study baseline | Pre-Day1 dosing baseline | Mean Baseline | Post-Day1 dosing | Post-Day5 dosing | Post-Day8 dosing |
| --- | --- | --- | --- | --- | --- | --- | --- |
| 50 mg/m², alternating weeks | DL1-1 | 2.48 | 12.00 | 7.24 | 2.74 | 2.03 | 7.24 |
| 50 mg/m², alternating with weeks of QOD dosing | DL1-2 | 1.97 | 29.00 | 15.49 | 3.17 | 7.63 | 1.08 |
| 50 mg/m², alternating with weeks of QOD dosing | DL1-3 | 3.50 | 2.77 | 3.14 | 1.03 | 0.70 | 2.90 |
| 50 mg/m², alternating with weeks of QOD dosing | DL1-4 | 2.85 | N/A | 2.85 | 1.31 | 4.90 | 2.30 |
| 50 mg/m², alternating with weeks of QOD dosing | DL1-5 | 1.97 | N/A | 1.97 | 0.12 | 0.15 | 0.19 |
| 50 mg/m², During Weeks 1-6 | DL2-1 | 2.59 | 3.88 | 3.24 | 1.42 | 1.42 | 0.68 |
| 50 mg/m², During Weeks 1-6 | DL2-2 | 3.21 | 4.03 | 3.62 | 1.29 | 0.32 | 0.11 |
| 95 mg/m², During Weeks 1-6 | DL3-1 | 2.23 | 2.76 | 2.50 | 2.48 | 6.31 | 4.92 |
| 95 mg/m², During Weeks 1-6 | DL3-2 | 2.68 | 47.10 | 24.89 | 3.42 | 0.55 | 0.04 |
| 95 mg/m², During Weeks 1-6 | DL3-3 | 2.88 | 2.85 | 2.87 | 1.89 | 1.63 | 2.75 |
| 130 mg/m², During Weeks 1-6 | DL4-1 | 1.08 | 0.16 | .62 | 0.26 | 0.41 | 0.48 |
| 130 mg/m², During Weeks 1-6 | DL4-2 | 2.21 | 0.05 | 1.13 | 0.38 | 0.33 | 1.08 |
| 130 mg/m², During Weeks 1-6 | DL4-3 | 3.17 | 0.21 | 1.69 | 0.61 | 2.47 | 16.50 |
| 130 mg/m², During Weeks 1-6 | DL4-4 | 4.09 | 0.18 | 2.14 | 0.16 | 0.43 | 0.50 |
| 130 mg/m², During Weeks 1-6 | DL4-5 | 3.75 | 18.90 | 11.33 | 10.60 | 3.75 | 1.49 |
| 130 mg/m², During Weeks 1-6 | DL4-6 | 2.83 | 1.31 | 2.07 | 0.31 | 0.06 | 0.00 |
| 160 mg/m², During Weeks 1-6 | DL5-1 | 5.19 | 5.90 | 5.55 | 0.96 | 5.27 | 5.59 |
| 160 mg/m², During Weeks 1-6 | DL5-2 | 2.65 | 38.70 | 20.68 | 7.75 | 0.13 | 5.55 |
| 160 mg/m², During Weeks 1-6 | DL5-3 | 3.71 | 3.99 | 3.85 | 0.95 | 1.86 | 0.95 |
| 160 mg/m², During Weeks 1-6 | DL5-4 | 1.74 | 5.37 | 3.56 | 2.25 | 0.31 | 3.37 |
| 160 mg/m², During Weeks 1-6 | DL5-5 | 4.46 | 3.71 | 4.09 | 6.62 | 5.28 | 3.25 |
| 200 mg/m², During Weeks 1-6 | DL6-1 | N/A | 2.10 | 2.10 | 0.88 | 3.75 | 0.40 |
| 200 mg/m², During Weeks 1-6 | DL6-2 | 2.67 | 0.16 | 1.42 | 2.45 | 3.61 | 2.17 |
| 200 mg/m², During Weeks 1-6 | DL6-3 | 2.58 | 13.90 | 8.24 | 17.90 | 16.90 | 14.70 |
| 200 mg/m², During Weeks 1-6 | DL6-4 | 3.14 | 0.01 | 1.57 | 0.00 | 0.01 | 0.01 |
| 200 mg/m², During Weeks 1-6 | DL6-5 | 2.40 | 3.29 | 2.845 | N/A | 6.11 | 5.81 |
